# Still Searching: A Meta-Synthesis of a Good Death from the Bereaved Family Member Perspective

**DOI:** 10.3390/bs7020025

**Published:** 2017-04-25

**Authors:** Kelly E. Tenzek, Rachel Depner

**Affiliations:** 1 Department of Communication, University at Buffalo, The State University of New York, Buffalo, NY 14260, USA; 2 Counseling, School, and Educational Psychology, University at Buffalo, The State University of New York, Buffalo, NY 14260, USA; rmpeloqu@buffalo.edu; 3 Center for Hospice and Palliative Care, Cheektowaga, NY 14227, USA

**Keywords:** end of life communication, family, good death, bereavement, presence

## Abstract

The concept of a good death continues to receive attention in end-of-life (EOL) scholarship. We sought to continue this line of inquiry related to a good death by conducting a meta-synthesis of published qualitative research studies that examined a good death from the bereaved family member’s perspective. Results of the meta-synthesis included 14 articles with 368 participants. Based on analysis, we present a conceptual model called *The Opportunity Model for Presence during the EOL Process*. The model is framed in socio-cultural factors, and major themes include EOL process engagement with categories of healthcare participants, communication and practical issues. The second theme, (dis)continuity of care, includes categories of place of care, knowledge of family member dying and moment of death. Both of these themes lead to perceptions of either a good or bad death, which influences the bereavement process. We argue the main contribution of the model is the ability to identify moments throughout the interaction where family members can be present to the EOL process. Recommendations for healthcare participants, including patients, family members and clinical care providers are offered to improve the quality of experience throughout the EOL process and limitations of the study are discussed.

## 1. Introduction

The thought of a good death seems contradictory in nature, but it continues to be researched and is of great value to healthcare participants. Hospice and end-of-life (EOL) care can be considered a paradox in that there is the possibility of beauty and quality living at EOL [1]. Paradoxes in EOL care can also come in the label of a good death, differences in desires for the EOL process and the idea that such a monumental moment for some is just another day for others [2]. There is a growing body of both quantitative and qualitative research on the topic of a good death [3,4,5], including work on a Good Death Inventory [6] and a Quality of Dying and Death scale [7]. Research on quality of death has focused on the perspective of clinical caregivers [8,9], the dying person [10,11] or a combination of healthcare participants [12,13,14,15]. Although there is not full consensus in the literature about what factors or qualities contribute to a good death, there are clear overlapping aspects to a good death, including “pain and symptom management, clear decision making, preparation for death, completion, contributing to others and affirmation of the whole person” [12] (p. 825). Many studies continue to find similar results that overlap with these categories [11,16,17]. Good death studies have also been conducted across disparate geographic locations, including rural developed and developing countries and different cultures such as Taiwanese widows, bereaved family members in Singapore, European countries, Japan, Kenya and Israeli community members [16,17,18,19,20,21,22].

Recently, two reviews of good death studies were published that focused on healthcare providers, patients and family members as they identified aspects of what a good death looks like [13,17]. The results illustrate consensus on certain ideas such as the importance of pain management, but there were also differences that indicate a highly individualized definition of a good death that included socio-cultural factors that defined quality of life [13,17]. Both reviews concluded with a call for more research [13,17]. More, specifically there was a call for healthcare participants at end of life to engage in more open, public dialogue surrounding death and dying [13]. In recent decades, research and clinical perspectives have shifted in focus with regard to viewing death as a pathological or medical phenomenon towards taking a more holistic and comprehensive perspective that values the positive aspects of death and dying [23]. This reconceptualization moves beyond the medical model of death and focuses on helping patients die at home and better quality of care in hospitals [24]. Recently, results of a systematic literature review illustrated that voices of rural participants at EOL have been marginalized and require attention because their needs are unique and present both challenges and benefits regarding the EOL experience in a rural setting [25]. Based on the results of this particular study, it is necessary for healthcare providers to understand the informational and medicinal needs of patients and family members in rural settings through communication and adequate dissemination of information [25]. While there are diverse contexts for EOL, it has been has considered a taboo topic, difficult for healthcare participants to bring up and engage in conversation [26]; if family members and patients know that the EOL is approaching, they have the opportunity to have meaningful conversations that promote a good death [27]. Therefore, we were interested in narrowing the focus to bereaved family member perspectives on going through the EOL process for a loved one. In doing so, we extend the EOL experience to continue to look past moment of death into bereavement experiences for survivors.

Family members have been noted to play numerous important roles with regard to the dying process as well as after the death of a loved one [25,28,29]. When an individual dies, it impacts the entire family and is noted as one of the most stressful life situations with regard to the family unit [30]. Additional research has found that quality of death may play a role in the manifestation of complicated bereavement. Particularly in a group of bereaved people, three aspects of quality of their loved one’s death were associated with later complicated bereavement: (a) dissatisfaction with the explanation to the family about the patient’s expected outcome; (b) the unreasonable cost of care; and (c) the family’s perception that the deceased person had not achieved a sense of completion about his or her life [31]. This research highlights the unique relationship between how a person dies and what potential impact that death may have on his or her family. Furthermore, the role of communication has been noted as critical in EOL care and the grief and bereavement process [32,33]. While the line of inquiry specifically related to a good death from a family perspective is relatively new, more research is needed to understand the family perspective at end of life. Research with family members who experienced loss noted several comprehensive factors that contribute to quality of death. These include personal, relational, biomedical, psychological and spiritual factors [3].

We seek to continue this line of inquiry related to a good death by conducting a meta-synthesis of published qualitative research studies that examined a good death from the bereaved family member’s perspective. A meta-synthesis allows for a more comprehensive understanding and conceptual or theoretical development that is not possible in one study alone [34,35]. We set out to engage in a systematic approach to research that included a literature search, description and screening for inclusion and exclusion criteria, quality assessment, data extraction and analysis [36,37,38]. Results of the meta-synthesis will be shared and discussed to better understand how family members experience the EOL process and the death of a loved one.

This meta-synthesis contributes to ongoing scholarly research and conversations related to EOL and a good death in three ways. First, we narrow the focus specifically to the perspective of the family members. Second, we expand the database search for articles, building upon the previous review [13], such that it is more inclusive of published articles. In doing so, we obtain a more comprehensive view of the literature. Finally, we will present a conceptual model in order to suggest future interventions that may be developed in order to help improve communication at EOL.

As our conceptualization of death continues to change towards a natural, developmental aspect of life, we must consider the communication processes within the family. No person dies in a vacuum and there are positive implications for reducing stigma, focusing on family bereavement interventions and encouraging more public dialogue for future family generations. Therefore, the guiding questions for the meta-synthesis were: What are bereaved family caregivers’ experiences of going through EOL with a loved one, and how does the EOL experience contribute to a good or bad death?

## 2. Materials and Method

Meta-synthesis was used in this study to answer research questions regarding family caregivers’ experiences of going through EOL and how that experience may contribute to a good or bad death. A meta-synthesis is a commitment of time and labor, but is beneficial for healthcare policy and practice [39]. We engaged in a four-step process for meta-synthesis that included (a) a literature search, (b) a quality appraisal, (c) classification and (d) synthesis [38,40].

### 2.1. Step 1: Literature Search Process

A comprehensive search was conducted, guided by key terms and the use of key databases. Then we eliminated duplicates and applied general search parameters to eliminate irrelevant search results. Once we had narrowed the results according to outlined general parameters, we prepared for full article review to determine study eligibility based on inclusion and exclusion criteria. The entire process is outlined below.

First, in an effort to expand the database search beyond [13], we included 14 databases for our search. Based on our guiding question for the study, we independently began our search using (a) PsycINFO, (b) Academic Search Complete, (c) MEDLINE with Full Text, (d) PsycARTICLES, (e) Psychology and Behavioral Sciences Collection, (f) PsycTESTS, (g) Social Sciences Full Text (H.W. Wilson), (h) Social Work Abstracts, (i) SocINDEX, (j) ALT Healthwatch, (k) CINAHL plus with full text, (l) Communication and Mass Media Complete, (m) Eric and (n) Health Source Nursing/Academic Edition. We carefully considered the key terms for our search rooted in relevant literature in the area of a good death and constructions of family. Therefore, examples of key terms included various combinations of: good death, end-of-life, family, bereavement, qualitative, hospice, quality death, caregivers and spouse. We independently searched for articles using the key terms through the databases outlined above and then came together multiple times to eliminate duplicates. Then we prepared for the next step, which was to set up the general parameters for our search and narrow down relevant articles that identified the topic of a good death, within the population of bereaved family members that had no restrictions on date or bereavement time. Our goal was to research a specific methodological approach to the EOL process, which was the “how”, research was done and we looked at qualitative studies [38]. It was at this point that we also eliminated other meta-syntheses, article reviews, commentaries, editorial and dissertations. Furthermore, articles had to be in English.

#### 2.1.1. Inclusion vs. Exclusion

Next, we met multiple times and discussed elements to guide the decision-making process for inclusion and exclusion criteria. In order to be included in the study, the articles were reviewed in full to identify the necessary components: (a) family members or loved ones of someone who was engaged in the dying process and/or passed away. The key here was the participant had to experience the loss of a loved one and the study was about that experience as opposed to a hypothetical loss; (b) mention good death at least once in the paper; (c) qualitative analysis/mixed methods were acceptable as long as the qualitative portion was clearly articulated; (d) the analysis had to provide results that portrayed the family perspective. For articles that combined healthcare participants, the family perspective had to be clearly articulated and represented equally in the analysis. If multiple healthcare participants were included and there was no differentiation between perspectives or there were not sufficient examples of family (i.e. quotes from the family were not equally represented among other perspectives) the article was excluded; (e) studies must have been reviewed and approved by an institutional review board. For our purposes, it was acceptable if a published study clearly articulated that an ethical review was conducted by an organization. We believed that, because of the enormity of the delicate time and topic in a family member’s life, we wanted to ensure the study was held to the highest ethical standards. As we were going through the process, if we were unsure, we discussed the individual article together and came to a decision. While some studies claimed consent was gained, we felt this was not enough information to meet this criterion and therefore studies were not included; (f) finally, to be considered for inclusion, studies had to be published in a peer-reviewed journal outlet.

#### 2.1.2. Search Results

**Figure 1:**
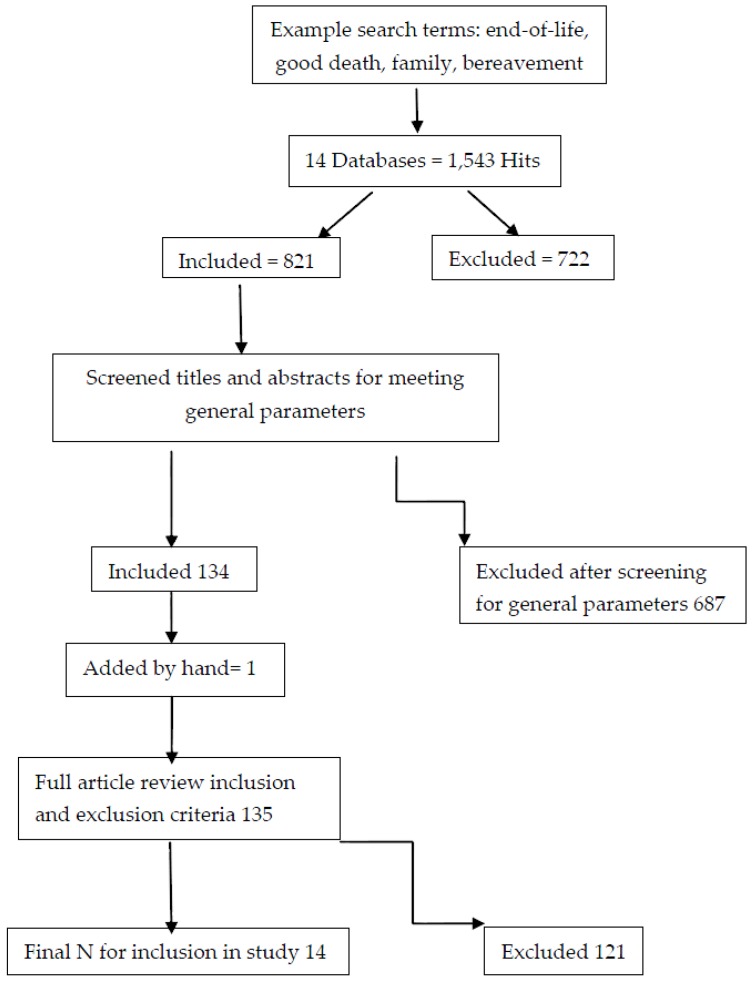
Search Results.

Step 1 of the process is shown in Figure 1. The results from our independent search efforts, from October 2016 to the end of January 2017 combined, returned 1543 hits. Once all duplicates were removed there were 821 remaining hits. At this point, we began the title and abstract search according to the general parameters outlined above, which resulted in 134 articles for full review. We noticed that a common article in the literature surrounding good death did not appear in our search, so we manually incorporated that into our search, which then changed our total number of articles to 135 for full review. After full review, 14 articles remained after we eliminated 121 articles. See Figure 1.

### 2.2. Step 2: Quality Appraisal

In order to address quality appraisal, we created a Microsoft Word coding document based on relevant criteria that examined the design and implementation of research using standards outlined by scholars [38,40,41,42]. We met to discuss each aspect of the coding sheet for quality appraisal and went through examples together until we had a shared understanding of what we believed quality to look like. We rated each article across key areas of demographic information, literature review, methodology, results and discussion. After completing the process for each article, we rated the quality of each article (e.g. 3 = high quality, 2 = moderate quality and 1 = low quality). As argued in previous research, if the article met the criteria for inclusion, they were not thrown out of analysis for low quality appraisal score [38,40].

### 2.3. Step 3: Classification Findings

Data were extracted by pulling information from the article to capture participant demographics and methodological details such as qualitative method, location of study, number of participants etc. We then also pulled the information directly from the results section from each article and placed this information into a Microsoft Word document to create our data set for coding purposes. We were able to then classify according to the Sandelwoski and Borroso system (no finding, topical, thematic, conceptual/thematic description, interpretive explanation) [38,43]. The results of this process are presented in Appendix A (Table A1).

### 2.4. Step 4: Data Abstraction and Synthesis

We were guided by [38]’s approach to meta-synthesis. In addition, while they argue that if classification results were no finding, topical, or thematic survey, then a meta-summary would be the more appropriate method, we argue, for the purposes of our study and moving scholarship forward, that an interpretive approach was appropriate. We wanted to begin to better understand bereaved family perspectives on a good death as a larger part of the EOL process and healthcare system. Furthermore, because there were no studies classified as no finding, only two were topical and a majority of studies were thematic, when the nature of qualitative work is to understand and make sense of phenomena through exploration and interpretation, an opportunity to truly integrate findings as opposed to comparing studies was presented [38].

**Figure 2:**
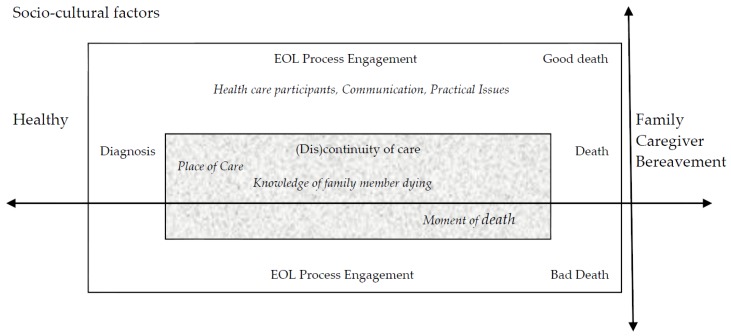
The Opportunity Model for Presence during the EOL Process.

We approached the data synthesis by combining taxonomic and constant targeted comparison grounded theory analysis [38,40]. We had not initially intended to use event timeline as an analytical tool, but because the conceptual elements aligned temporally, we also incorporated timeline into our analysis [36]. We followed a grounded theory approach by Charmaz [44]. The process entailed reading through the data to familiarize ourselves with the data and then began opening coding. Next we engaged in the processes of focused and axial coding, followed by diagramming. The results of this iterative process are showcased in the conceptual model presented in Figure 2.

### 2.5. Validity

As outlined by meta-synthesis scholars, the rigor and validity of the meta-synthesis is important. We took the following steps: (a) a detailed and thorough literature search; (b) collaborative discussion on appropriate inclusion and exclusion criteria; (c) quality appraisal by creating coding sheets guided by previous research; and (d) an audit trail of the collaborative process [38,40].

## 3. Results

Results of the meta-synthesis included 14 articles and are presented in Appendix A (Table A1). The 14 articles had quality appraisal scores ranging from 14 to 21, with an average score of 17.7. Results of the classification findings included eight thematic articles. Furthermore, three were conceptual, two were topical and one study was interpretive. None of the studies were classified as no findings. Results point to a complex and complicated process related to constructions of the EOL experience. There were a total of 368 participants that experienced a range of bereavement lengths when all studies were combined. The research questions that motivated the meta-synthesis were: what are bereaved family caregivers’ experiences of going through EOL with a loved one, and how does the EOL experience contribute to a good or bad death? The results highlight that interpretation of death of a loved one included both unique and specific moments of care and overall recollections of the dying process. Ultimately, we observed that the EOL experience is influenced by multiple intertwined factors that may influence interaction at each new turning point or change within the patient’s narrative as recalled by the family caregiver. When looking at the findings of the 14 studies as a whole, we found that the family caregivers who experienced positive engagement at one point or throughout the entire process had a better EOL experience. Conversely, family caregivers who experienced a negative moment(s) within the EOL care process expressed negative experiences. Based on the idea that there were critical moments recalled by family members that characterized the EOL experience and consequent labeling of a good death, we present a conceptual model, called *The Opportunity Model for Presence during the EOL Process*.

Analysis of the 14 studies taken in concert illustrated a progression through the dying experience as recalled by family caregivers. Therefore, we argue that a timeline emerged based on family caregiver’s memory of events, starting from their loved one being healthy (not clearly dying, no idea it was a possible health outcome) continuing through the death of his or her loved one and into bereavement (see Figure 2). We do not mean to say that the timeline is linear and the events take place in the same order for all healthcare participants. Rather it is a dynamic timeline that fluctuates according to the EOL experience. At each stage of the timeline, there is an opportunity for engagement that allows the family member to be present to the EOL experience. When the opportunities for engagement are missed, the positive moments and experiences for quality and satisfying EOL care are also missed. When this is the case, we argue negative experiences and low engagement result in labeling the experience as a bad death, leading to complicated bereavement.

Figure 2 illustrates a timeline that is immersed in (a) socio-cultural factors [19,20,45,46,47], and as the healthcare events and experiences progress, two themes emerge; (b) levels of engagement [19,45,48,49,50,51]; and (c) (dis)continuity of care [19,45,50,52,53]. These contribute to the EOL experience, which then is labeled as good or bad. The EOL experience does not end there however, as it extends into after-death experiences and shapes the bereavement process [45,48,50,54]. The results presented here compose a conceptual model that integrates diverse experiences throughout the EOL process. We remained grounded in the data as conceptual linkages emerged and we attempted to capture incredible nuance that showcased individual differences, but also provided clarity on elements that lead to a positive or negative EOL experience for family carers.

### 3.1. Socio-Cultural Factors

Based on the studies we defined socio-cultural factors as the different values, cultures, preferences and experiences that make up the environment for healthcare interactions. We include individual differences, patient-caregiver belief systems and past experiences in the socio-cultural factors. For example, definitions of EOL and bereavement experiences are incredibly diverse and different based on the family caregiver’s experience, knowledge and media exposure [20,45,48,52].

Additionally, components of the socio-cultural elements that shape our environment include biomedical, psychosocial and spiritual components [19,48,55,56]. Finally, we argue language is part of the socio-cultural environment that healthcare participants bring with them into the EOL process. The use and understanding of language in part defines the environment. For caregivers, when they entered into the EOL process for their patient and interacted with doctors, the use of medical terminology worked socioculturally for the doctor in the medical context, but it did not work for the caregiver, who did not have the same language knowledge and experience [47]. The socio-cultural elements that make up peoples’ environments are then carried with them as the timeline shifts from “healthy” to “diagnosis”. The next section continues to discuss the EOL process from “diagnosis” to “death”. We will first focus on the theme of EOL process engagement and subsequent categories of healthcare participants, communication and practical issues.

### 3.2. EOL Process Engagement

The theme of EOL process engagement identified the characters involved in EOL care known as healthcare participants, the type of interpersonal interactions that take place through communication and organizational or systematic issues that have the potential to influence how EOL care is perceived, which we refer to as practical issues.

#### 3.2.1. Healthcare Participants

When the interaction moves forward into the healthcare scenario from healthy to initial diagnosis, certain participants entered into the EOL process in different roles. Clinical staff including doctors, nurses, hospice aides etc. began to fulfill clinical care needs. The familial healthcare participants include, spouses, children, siblings and grandparents who enact certain roles. For family members, that role could extend into healthcare provider or patient advocate. Some fulfilled this specific role because it was the morally appropriate thing to do [45,52,54]. There were also community members and religious or spiritual care providers who offered a certain type of care at EOL [19,49].

For family carers and participants in the studies, when care was needed and if it was administered and received properly (i.e. a smooth integration of care by provider), family carers felt good about the situation and were empowered [45,52,53]. This was also seen in family caregivers’ desire for accommodation from medical staff/care facility to meet patients’ needs. This also had to do with the carer knowing their loved one’s history and preferences. One article illustrated that they had little understanding of how the medical care team worked. When working with specialized care teams, the roles were extremely complex [51]. When there was conflict between medical staff and family caregivers, the outcome was often feelings of frustration, isolation and powerlessness. For example, “if care was viewed as high quality, participants described a willingness to entrust the dying person’s care to professional caregivers. If they were insecure about the quality of care, trust was lacking and they reported a need to be on guard as the dying person’s advocate” [54] (p. 910). Role clarity was complicated by blurred lines between professional clinical caregiver roles and family caregiver roles, especially when the clinical staff had an expectation/assumption that the family caregiver knew and understood the biomedical nature of EOL processes. A thread that underlies the healthcare participant role and provision of care is communication. The next section delves into defining and explaining the communication portion of engagement during the EOL process.

#### 3.2.2. Communication

We argue communication, or the interaction among healthcare participants during EOL care, is at the heart of the *Opportunity Model for Presence during the EOL Process*. Communication was comprised of elements such as social support, decision-making and relationships. Characteristics of interpersonal communication included trust, intimacy, empathy, trust and listening. The studies indicate a wide array of communication exchanges and experiences, and what we observed was that with the instances where the family caregiver perceived to be listened to, received kindness, social support and engaged in decision-making with other healthcare participants, the EOL process at that moment was positive [19,48,51,55]. When descriptions of healthcare encounters included a lack of involvement in decision-making, lack of listening, no kindness or empathy, family caregivers were frustrated, upset and had a negative perception of that EOL experience [47,50,52,54,56]. We argue here again that the negative examples due to communication were missed opportunities for a positive experience that can be addressed. The third component of EOL process engagement is practical issues throughout the EOL process.

#### 3.2.3. Practical Issues

Practical issues emerged from the data set as tangible, concrete obstacles that influenced the quality of EOL process perceived by family caregivers, including spatial attributes, after death management and access to care and cost. First, in terms of spatial attributes, participants mentioned the set of hospital rooms, cleanliness of facilities and how these influenced the privacy or lack thereof during the EOL process [19,49]. We often think of the next step after death as funeral arrangements or cremation, or even grief and bereavement, but there are practical aspects that family caregivers mentioned they were not aware of and it influenced the EOL process immediately after their loved one had died [45]. Finally, there was not a strong thematic representation of access to care and care within the data set, but we argue it is such an important issue that it warranted mention as a practical barrier to even entering into the preferred type of EOL care and management of accessing health care and payments [54]. As we move both through the event timeline and concurrently address the sociocultural factors and levels of engagement, we now move deeper into the EOL process and address the theme of (dis)continuity of care. This theme has three categories that have the potential to oscillate back and forth between place of care, knowledge of family member dying and moment of death. We continue to thread the sociocultural factors through the model combined with engagement factors that simultaneously permeate the inner core of the model.

### 3.3. (Dis)continuity of Care

In the section of the model that addresses (dis)continuity of care, the data indicated that there was the potential for a lot of movement in the progression of the EOL process. Therefore, the overarching theme here is related to continuity of care through the process and identification of moments where there was either a smooth progression of care provided throughout the EOL process or a break in continuity, resulting in uncertainty or dissatisfaction. The section below discusses the categories of (dis)continuity of care.

#### 3.3.1. Place of Care

Results of the analysis illustrate that the place of EOL care was a component to the EOL process [48,50,52,56]. Examples of place included: home, hospice, hospital and tertiary care facility. It was also evident that family caregiver experiences of place of care involved a combination of facilities and transfers among places of care or the missed opportunity to transition to a different type of care. Several studies noted that EOL care discussions occurred too late or were too ambiguous, which led to their loved one dying prior to receiving the change in care [56]. This, at times, created stress for the family caregiver because they realized their loved one could have died in a different way or in a different setting. The place of EOL care was noted and linked to the type of care accessed (dying at home versus dying in a hospital) and subsequently utilized (whether a transfer was discussed, secured or not addressed), as well as the family member’s overall perception of the dying process. The last-minute nature of EOL discussions relating to plan of care required flexibility in that, even if the desires of patient were known, the biomedical needs of that patient may shift and change options for receiving EOL care [52,56].

#### 3.3.2. Knowledge of Family Member Dying

The place of care was complicated by family caregiver knowledge of where their loved one was in the dying process. There is a range of possible combinations for knowledge and understanding of the reality of the loved one dying. For example, the family may not know their loved one was at EOL and it was a complete shock. This may be because they were not told of the biomedical status of patient [50] or they may have been told, but they did not/could not accept that. Another possibility could be that the family member knew and it was communicated so much that it became offensive. Another study noted the importance of preparing for the death of their loved one through awareness and understanding, indicating it is best if patients and family members know and understand that their loved one is dying [45]. The data also note the importance of the family members having anticipatory time to reflect on and begin to make sense of their loved one’s death prior to the moment of death, compared to individuals that had an ambiguous understanding of their loved one’s situation who reported the moment of death as more traumatic or complicated due to the unexpected nature of the situation. The place of death and knowledge further complicated the moment of death.

#### 3.3.3. Moment of Death

While (dis)continuity of care is the primary theme for this portion of the conceptual model, we know that these factors are oscillating and we see the categories of place of care and knowledge level go back and forth as the loved one’s health changes. This extends into the moment of death. Here we see conflict arise when a patient did not die in the place they wanted, the family caregiver was unaware that their loved one was dying and when a family caregiver was not present for the death of a loved one. While a family member could not have knowledge regarding the imminent death, this also means that they were not prepared for the actual moment of death and this shaped the experience of losing a loved one. The biomedical option of sedation was also discussed as an option for EOL care that may be administered for patients in different physical locations at various points in the EOL process, but when death was near, it could complicate the exact moment of death. This example had particular relevance to understanding the palliative sedation intervention, which can lead to distress [20]. If we continue along the timeline enmeshed with sociocultural and EOL engagement and proceed until after the moment of death, we arrive at this reflective space of labeling the EOL experience ranging from a good death with high engagement to a bad death with low engagement.

### 3.4. Good vs. Bad Death Experiences

Based on our findings, while a good death had to be mentioned at least once in the paper, not all studies used the label good or bad death to describe their experiences. What we see are examples of peaceful death, beautiful death, death quality etc. [46,49,55] as well as traumatic or stressful deaths [45,48,50,53,54,55]. What our model illustrates is that the entire process is made up of many turning points or moments of opportunity. These moments of opportunity are particularly salient when the family caregiver felt there were barriers to care, a discontinuity of care, if the death came as a shock or if the patient’s needs were not fulfilled, because this left family caregivers with negative feelings of low engagement and powerlessness [48,49,50,51,53,54,56]. Once the death has happened, the timeline pushes out of engagement and (dis)continuity of care into family carer bereavement.

Results illustrate that it is an incredibly difficult experience to lose a loved one. Authors of one of the studies conceptualized the grief trajectory as including three main stages: (a) the initial loss or gap left from the death of a loved one; (b) the acute or intense grief following the death; and, finally, (c) a stepped journey towards bereavement recovery [56]. The dynamic and overlapping nature of both the end-of-life process and bereavement of the family member can be difficult to disentangle from one another. However, it became clear how important it was for family caregivers to spend the EOL engagement and (dis)continuity of care portions of the timeline present with the loved one, being active and living to the fullest at EOL. Therefore, the final section discusses the overarching conceptual component that drives the theoretical model: presence.

### 3.5. Presence

Based on the studies, we define presence in part as being physically present with the patient, if that was desired, and, more holistically, being focused and aware [19,46,49,53,55] of what was happening at each step in the timeline. We do not mean that being physically present for every family member will determine a positive or negative EOL experience. However, being present and aware of what patient wanted along with knowing what options were available to the patient and family become necessary so that an informed decision can be made. This creates positive opportunities for high engagement. We argue when the family member had the opportunity to be present and engage in a variety of ways, by (a) awareness of the illness’ trajectory, (b) saying an appropriate goodbye, (c) negotiating the plan of care with the clinical staff, (d) being an advocate for the patient and (e) experiencing kindness, empathy and support, then a quality EOL experience was possible [20,45,49,53,54]. These opportunities for presence do not happen in isolation. Being fully present to the EOL experience requires collaboration with all healthcare participants and an awareness of sociocultural factors and engagement levels. Finally, we argue that the bereavement experience is cyclical and loops back to the beginning of the timeline healthy, when the caregiver begins the process again for a loved one or they receive a diagnosis and enter into the process model. The relational nature of death, dying and loss cannot be fully explicated from one another. Each represents an opportunity for the reconceptualization of these experiences. This is particularly noted by one of the articles as the authors write that “bereavement was found to be an individualized, contextualized and multifaceted experience… with subthemes of positive assistance or of being hampered by factors both before and after death” [48] (p. 263). These results stress the importance of expanding the conceptualization of bereavement to include an anticipatory period that encompasses the cyclical and cumulative nature of grief after the death of a loved one.

## 4. Discussion

The guiding questions for this meta-synthesis were (a) what are bereaved family caregivers’ experiences of going through EOL with a loved one and (b) how does the EOL experience contribute to a good or bad death? Results indicate that the experiences of caregivers going through the EOL process with a loved one are incredibly mixed. There were both good and bad experiences, good moments and terrible narratives of the poor communication that left a negative imprint on the family member’s EOL experience and grieving process. In terms of what components made up the good death experience, the results closely align with previous scholarship in the good death literature, including pain and symptom management, clear decision-making, preparation for death and communication [6,7,12,14,17,57,58]. Furthermore, the results align with an earlier meta-synthesis of EOL care from the family perspective that reported the necessity of quality communication among healthcare participants and clinicians being open about imminent death [30]. While the review discussed the importance of advance care directives to the feeling of being prepared, advance care planning was not strongly represented within our data set. However, we believe that the process nature of EOL and making changes to the sociocultural factors, such as normalizing death through advanced care directives, can really improve the care provided throughout the EOL process. The importance of support throughout the entire process is crucial, including continued support after the loss of a loved one, as times of grief and sadness require a reconstruction of life without the loved one [45,48,59]. Although loss is challenging in any circumstance, we argue the loss of a child is extremely difficult and changes the lives of the surviving bereaved [60]. While much of the good death literature focuses on the natural progression to EOL, the importance of bereavement when parents and siblings lose a loved one, the biopsychosocial and spiritual factors, communication and decision-making are even more important because the bereavement experience becomes part of the sociocultural factors that will influence the family’s next experience with EOL.

It became clear that no one entered into the diagnosis phase and the EOL process intending to have negative experiences, but nonetheless this was often the case. We argue that with the use of the *Opportunity Model for Presence during the EOL Process*, if family members are aware of the process of EOL then they can be more aware of being present to and advocating for the care their loved one desires at EOL. As previously stated, we believe that quality communication is essential for providing and experiencing care at EOL. As the following quote explains, it is not just about dying, but living throughout the entire process: “The pivotal role of good communication is the route to ensuring that issues are addressed, with hope maintained for the patient to live as well as possible until they die, and that patients’ quality of life is maximized” [61] (p. 91). If we can maximize the quality of life while the patient is alive with the knowledge, understanding and acceptance that the loved one will pass away, all of the healthcare participants can become engaged, present and continue to make memories. These memories become what the family member will have with them after their loved one dies. We do not imply this is an easy process, but we do emphasize the importance of capturing the opportunities to be present throughout the EOL process will have a positive influence on bereavement.

Recommendations are offered in the latest Institute of Medicine report to continue to address barriers to quality and individualized EOL care including (a) care delivery, (b) clinician-patient communication and advance care planning, (c) professional education and development, (d) policies and payment systems and (e) public education and engagement [62] (p. 3). While we believe each of these aspects can be applied to the current study, we want to offer two recommendations for moving research in the area of EOL processes. We argue that specifically related to concepts of a “good death”, family caregivers and bereavement in this study align with the recommendations from IOM for communication, professional education and development and public education and engagement. First, with the model, we propose there are areas for interventions to be developed to help healthcare participants communicate about EOL. Research illustrates that healthcare participants struggle with engaging in EOL conversations [26,62,63]. Methods and models currently exist to help clinicians interact with patients and family members: SPIKES [64], COMFORT [65], PREPARED [66] and BATHE [67]. We argue these conversations need to continue to be integrated into medical education through curriculum development and continuing education for medical professionals, wherein the focus on palliative care and hospice become more prevalent. However, we do not mean to imply that the enormity of engaging in the EOL conversation should only fall on the doctors. Patients and family members need to be their own advocate as well throughout the EOL process and be involved in conversations related to advance care planning and team meetings [62]. We argue that being more open about experiences of losing a loved one in the past and integrating EOL conversations into conversations much earlier in the educational system would create the opportunity to naturalize EOL and continue the dialogue regarding death and dying [13]. We argue more classes at the college level, not only for medical students but as courses offered in communication, social work and nursing departments, would be extremely beneficial.

Secondly, while the timeline we propose in the model is not linear in nature, there is an opportunity for a “presence check” at all stages of the EOL process. According to the IOM report, achieving the goal of affordable care that is sustainable for people with advanced illness is possible and requires, “the provision of quality care that offers patients and families both compassion and choice” [62] (p. 22). As healthcare providers and scholars, we must continue to ask questions such as “how are the relationships, how is the communication, and what practical barriers are in the way of achieving the goals for EOL interaction?” This may be something as mundane as the cleanliness and ease of maneuvering the facility and greeting fellow healthcare participants with a smile. We realize that communication and relationships take work and there are certain organizational constraints, but the results of this meta-synthesis illustrate how meaningful and powerful the positive aspects of communication can be on EOL processes including listening, involvement in decision-making, appropriate touch and support throughout the EOL process. Familiar faces from diagnosis to bereavement seemed to be significant. By combining clinical competence through interpersonal communication skills and knowledge as defined by the IOM with “presence checks” for healthcare participants we can further provide quality care through compassion and choice [62].

Limited by search parameters, we intentionally searched broadly to capture articles that fit our search criteria, but then we also had specific inclusion and exclusion criteria that narrowed the scope. We feel this is both a boon and a bane when it comes to obtaining a holistic picture of the context we were searching to understand. Secondly, methodologically, a meta-synthesis calls for synthesis of findings within the articles, but without the use of original participant quotations. The meta-summary however, is more quantifiable and focuses on frequencies at a surface level of understanding and interpretation [38]. We felt that our articles for inclusion were more on the thematic side of the scale but, because of the rich nature of experiences and deeply emotional topic area, they warranted a more in-depth look beyond categorizing by frequencies of information analysis. Therefore, the conceptual model is proposed to help healthcare participants understand the process in front of them during EOL and create the opportunities for presence checks, but should be taken as something that needs further investigation for nuances that may have been lost in the meta-synthesis in order to see the bigger picture of good death and family bereavement. We want to continue research efforts to apply the model in future studies and create interventions at different timelines where the findings indicate there is a window of opportunity for high engagement.

## 5. Conclusions

We argue the meta-synthesis contributes to ongoing scholarly research and conversations related to EOL and a good death in three ways. First, we narrowed the focus specifically to the perspective of the family members. Second, we expanded the database search for articles from the previous review [38] to be more inclusive of published articles and obtain a more comprehensive view of the literature. Finally, we proposed a conceptual model in order to help healthcare participants have more positive EOL experiences. We look forward to continued efforts in this area of EOL communication to help healthcare participants, especially bereaved family members, achieve a good death.

## Appendix A

**Table A1:** Step 3 Classification of data.

Source/Country	Aim/Purpose	Methods/Data Collection	Sample N	Bereavement Period	Classification and QA Score
Holdsworth, (2015) [45] United Kingdom	“The aim of this article is to describe the end-of-life experience from the point of view of bereaved family carers with particular reference to the role that care providers play in shaping this experience” [45] (p. 835).	Interviews	45	6–10 months	Thematic/21
Wilson, MacLeod, & Houttekier, (2016) [48] Canada	“As it does not appear that research has focused on a linkage between death quality and the intensity of bereavement grief, we conducted a mixed-methods research study to determine if this relationship exists and for evidence-based insights into any connections between bereavement grief and death quality” [48] (p. 261).	Interviews	41	5 months–8 years	Thematic/18
Nelson, Schrader, & Eidsness, (2009) [54] United States	“The aim of this study was to explore end-of-life (EOL) experiences of South Dakotans who had experienced the death of a loved one in the last 5 years” [54] (p. 905).	Interviews	35	Within 5 years	Topical/14
Lee, Woo, & Goh, (2013) [19] Singapore	“The aim of this study was to examine the concept of a good death from the perspectives of both the dying person and the family caregiver, as perceived by bereaved family caregivers of advanced cancer patients” [19] (p. 37).	5 focus groups, 1 interview	18	6–18 months	Thematic/16
Kongsuwan, Chaipetch, & Matchim, (2012) [55] Thailand	“The purpose of the study was to describe the concept of a peaceful death in ICUs from Thai Buddhist family members’ perspectives” [55] (p.152).	Interviews	9	2–12 months	Conceptual/20
Abib El Halal, Piva, Lago, El Halal, Cabral, Nilson, & Garcia, 2013 [47] Brazil	“The aim of this study was to explore parents’ perspectives of the quality of the care offered to them and their terminally ill child in the child’s last days of life in two Brazilian PICUs” [47] (p. 496).	Semi-structured interview	15	6–12 months	Thematic/16
Donnelly & Battley, (2010) [49] Ireland	“To describe the contemporary experience of relatives in a tertiary referral hospital of the moment of death, traditionally a very significant event” [49] (p. 96).	Interviews	24	Unclear	Topical/18
Robert, Zhukovsky, Mauricio, Gilmore, Morrison, & Palos; (2012) [51] United States	“To understand the needs and experiences of bereaved parents whose child had received care at one National Cancer Institute-designated comprehensive cancer center. The investigators were particularly interested in the parents’ perceptions of the care received by their child, their expectations of palliative care, and recommendations on how best to improve palliative care for children with cancer and their parents” [51] (p. 318).	Focus groups	14	Lost a loved one a minimum of one year before study	Thematic/20
Evans, Cutson, Steinhauser, & Tulsky (2006) [52] United States	“To describe caregivers’ reasons for transfer from home hospice to inpatient facilities, preferences for site of care and death, and their experiences during these transfers” [52] (p. 100).	Interviews	18	Contacted about study at least four weeks after patient death	Thematic/17
Jack, O'Brien, Scrutton, Baldry, & Groves, (2015) [53] United Kingdom	“To explore bereaved family carers’ perceptions and experiences of a hospice at home service” [53] (p. 131).	Interviews	20	At least 3 months	Conceptual/20
Williams, Bailey, Noh, Woodby, Wittich & Burgio (2015) [56] United States	“The purpose of this qualitative study was to explore the personal and interpersonal context of next-of-kin’s discussions with clinicians regarding discharge planning to home hospice or inpatient palliative care service for hospitalized veterans” [56] (p. 51).	Participant obser-vation, focus groups, and interviews	78	3–6 months	Interpretive/17
Wilches-Gutiérrez, Arenas-Monreal, Paulo-Maya, Peláez-Ballestas, & Idrovo, (2012) [46] Mexico	“To ascertain the elements comprising the health/illness /death process in the context of a holiday in this municipality (Yautepec, Morelos, Mexico)” [46] (p. 775).	Interviews	7	Loss within the last four years	Conceptual/18
Bruinsma, Brown, van der Heide, Deliens, Anquinet, Payne, Seymour, & Rietjens, (2014) [20] Belgium, United Kingdom, Netherlands	“The purpose of the study was to explore relatives’ descriptions and experiences of continuous sedation in end-of-life care for cancer patients and to identify and explain differences between respondents from the Netherlands, Belgium, and the UK” [20] (p. 3243).	Interviews	38	3–18 months	Thematic/18
Workman & Mann, (2007) [50] Canada	“To identify areas for improvement in delivering high quality end-of-life care on the medical teaching unit” [50] (p. 433).	Interviews	6	6 months	Thematic/15
